# The Influence of Clinical Factors on Treatment Outcome and a Recurrence of Surgically Removed Protruded Subungual Osteochondroma and Subungual Exostosis

**DOI:** 10.3390/jcm12196413

**Published:** 2023-10-09

**Authors:** Mikołaj Dąbrowski, Damian Rusek, Aleksandra Dańczak-Pazdrowska, Anna Litowińska

**Affiliations:** 1 Adult Spine Orthopaedics Department, Poznan University of Medical Sciences, 61-545 Poznan, Poland; 2 Anmedica-Healthy Foot Center, Grunwaldzka Street 121, 60-313 Poznan, Poland; kontakt@anmedica.com; 3 Department of Pathomorphology, Regional Hospital in Poznań, Juraszów 7 St., 60-479 Poznan, Poland; d.rusek@onet.pl; 4 Department of Dermatology, Poznan University of Medical Sciences, 60-806 Poznan, Poland; aleksandra.danczak-pazdrowska@ump.edu.pl

**Keywords:** nail surgery, subungual exostosis, subungual osteochondroma, subungual tumor, subungual mass, clinical factors, gender, drill, burr, histopathology, complete surgery excision technique

## Abstract

Background: Subungual exostosis (SE) and subungual osteochondroma (SO) are benign solitary lesions that grow from the distal phalanx. The mass itself is typically painless, but pressure on the nail plate can result in pain and deformity of the involved digit. Tumors can be correctly diagnosed based on clinical, histological and radiographic appearance alone. Surgical resection of SE/SO is typically curative, with a small risk of recurrence. Methods: The study was retrospective and observational, involving 74 patients with subungual SE/SO. The surgical procedure consisted of the removal of the tumor from the dorsal approach under digital anesthesia. The procedure was assessed using a questionnaire and photo documentation after a minimum of 6 months after surgery. Results: A total of 85% of respondents were satisfied with the procedure. Nearly 80% of patients rated the cosmetic effect as good or very good. Young age and pain intensity after surgery showed statistically significant associations with worse satisfaction. Age < 18 was associated with recurrence. Conclusions: Worse satisfaction is strongly associated with recurrence. Gender, duration of symptoms, pain before surgery and tumor size and destruction of the nail plate had no significant effect on recurrence. The technique using burr appeared to be a more effective treatment.

## 1. Introduction

Subungual exostosis (SE) and subungual osteochondroma (SO) are uncommon, benign osteocartilaginous tumors of the distal phalanx of the toes or fingers [1]. A total of 75–80% of cases affect the dorsal or medial surface of the distal phalanx of the big toe [2,3,4]. Subungual osteochondroma most frequently affects children and adolescents, while subungual exostosis is more common in adolescents and young adults [5,6].

Possible triggers for SE and SO include trauma, tumors (reactive metaplasia resulting from microtrauma), infections, hereditary abnormalities and activation of cartilaginous cysts [3].

The most frequent symptoms of SE/SO include discomfort or pain caused by pressure on the nail plate, redness, mass/swelling of the nail, nail deformity, abnormalities in the nail bed or other complaints such as shoe wear rubbing or stiffness [3,7]. The symptoms affect the patient’s quality of life [3]. Histologically, SE has fibrocartilage, while SO has a layer of hyaline cartilage, although mixed types are also described. SO arises from the metaphyseal region of the distal phalanx and presents as a well-defined, circumscribed, pedunculated or sessile mass formed by the trabecular bone projecting from the distal phalanx. Contrarily, SE shows the trabeculated bone arising from the dorsal or dorsomedial aspect of the tuft, extending distally with no continuity between the lesion and the medullary cavity of the underlying bone. The lesion may have a broad base, extending upward with a narrow end or may be narrow-based with progressive widening [2,8,9].

Pathognomonic translocation t(X;6)(q22;q13–14) (insulin receptor substrate (IRS)-4 gene) was demonstrated in SE [10]. Hereditary multiple exostoses (HME) is a dominant genetic condition that is caused by a mutation in the genes EXT1 or EXT2 (important for the normal development of bones and cartilage) [11]. In solitary sporadic osteochondromas, homozygous deletions in EXT1 can be found in about 80% of tumors [2]. There are no reported cases of positive family history or malignant degeneration of the SE and SO [3]. In practice, it is difficult to distinguish SE from SO both clinically and histologically. A bone growth with a stalk-like shape that is visible on the back or back-side of the end of the toe bone can be diagnosed by x-rays. SO has a bone part that is connected to the toe bone [5,12,13]. SE demonstrates a bony outgrowth emanating from the dorsal or dorsomedial aspect of the distal phalanx and is typically not continuous with the underlying bone, contrary to SO. Radiographs often underestimate the actual tumor size due to the radiolucency of the fibrocartilaginous cap. Although these changes are usually visible on X-rays, in some instances, especially when small areas such as the distal phalanx of the toe are affected, cross-sectional imaging may be required [14]. Magnetic resonance imaging is most helpful in demonstrating the tumor juxtaposed with the surrounding structures. Theoretically, MRI can distinguish between SO and SE because the hyaline cartilage cap in SO has high signal intensity on T2-weighted images, whereas the fibrocartilaginous cap in SO is hypointense in all MRI sequences [14]. However, small structures/lesions may not be detectable by MRI, and the distal toe phalanx may warrant the use of computed tomography (CT) because it offers the advantage of tracing the origin of the lesion to the metaphysis or tuft [14].

There is debate among some authors about whether SE is a type of SO or a distinct condition. Others have pointed out some differences between them, including ultrasound assessments [3,7,13,15,16,17].

The role of nonoperative management is limited because the condition is generally progressive. Expectant management may be considered in asymptomatic cases, but most patients reported symptoms. If symptoms of SO/SE are present, complete surgical excision remains the gold standard treatment for these conditions regardless of the lesion type [5,13,18].

The surgical resection of SO and SE is typically curative, with a low risk of recurrence [3,4,5,13,15,19]. There are no significant differences in recommended management or recurrence rates between SE and SO [1]. Surgical techniques vary and the best procedure involves complete marginal excision with minimal trauma to the nail bed [3,20]. Rare recurrences can be managed by re-excision [8].

Despite their benign nature, these conditions often require surgical intervention to alleviate symptoms and improve patient outcomes. However, the success of surgical treatment and the risk of recurrence can be influenced by various clinical factors. Understanding these factors is essential for optimizing treatment strategies and improving patient care. 

The aim of the study was to evaluate the influence of clinical factors on treatment outcome and recurrence of surgically removed protruded subungual osteochondroma (SO) and subungual exostosis (SE).

## 2. Materials and Methods

### 2.1. Participants

Between June 2018 and November 2022, 74 children, adolescents and adults with subungual exostosis or subungual osteochondroma were treated surgically. The entire analysis is reported according to the STROBE checklist [21]. Participants represented a convenience sample identified through the outpatient surgical clinic at Anmedica-Healthy Foot Center (Poznan, Poland). All participants underwent the procedure as an outpatient day procedure approximately one to four weeks after their initial consultation with the same podiatric nurse. The procedure was performed in the operating room under local anesthesia. All procedures were performed by the same orthopedic surgeon with prior experience in this procedure. The first author has 10 years of experience in spine surgery (Wiktor Dega Hospital in Poznan, Poland) and orthopedic surgery and 6 years of experience in nail surgery (over 3500 surgical procedures). 

The inclusion criteria were: underwent surgical excision of SO or SE, histopathological confirmation of SO/SE and minimum follow-up of at least six months after surgery. The baseline assessment included questions regarding the duration of symptoms, risk factors and previous treatment. The surgeon also classified the severity of the disease using the III type described by Hao Li et al. [22]. Exclusion criteria were: unwilling or unable to provide informed consent or follow-up, not understanding Polish and significant medical comorbidities. In more than half of the cases, we were unable to distinguish SO from SE based on the clinical, histopathological and radiological features, therefore we did not divide the study group, which is also confirmed by other authors [1,3,15]. The use of CT for every patient without absolute indication was unjustified.

Clinical data, including sex, age, trauma history, infection history, nail appearance, lesion location, the relationship between lesion location and nail bed and surgical treatment processes were recorded. 

Written consent was obtained from the patient; for juvenile participants, written consent was obtained from the parent or legal guardian as well. The study was approved by the Bioethics Committee of the University of Medical Sciences, Poznań (No. 32/20).

### 2.2. Surgical Technique

#### 2.2.1. Preparation for the Procedure

**Figure 1:**
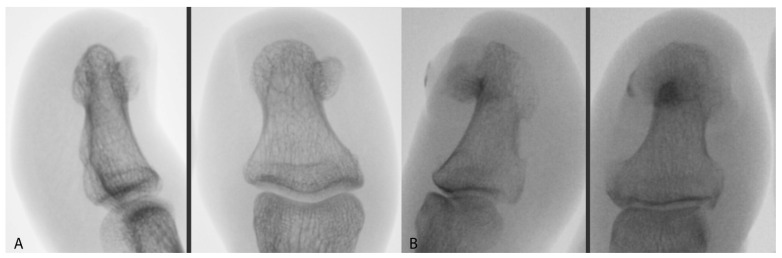
AP and lateral X-ray image of (**A**) SO and (**B**) SE.

In order to qualify for surgery, each patient received a health questionnaire, which was sent to the Healthy Foot Center along with photographs and X-rays (Figure 1). The patients received periprocedural recommendations: an inventory of dressing materials, a method of preparing the toe for surgery, as well as postoperative procedures, including instructions for changing the dressing. They also received recommendations that, for three consecutive days before the procedure, the toe should be soaked for 10 min in a prepared solution of potassium permanganate and then dried and lightly bandaged. On the day of the surgery, the patient reported with the toe secured by a bandage. 

#### 2.2.2. Anesthesia

First, the involved toe was prepared in a sterile fashion, and ring nerve block anesthesia was applied using 2% lidocaine without epinephrine. After the anesthesia procedure, an elastic tourniquet was placed at the base of the toe to maintain a clear and bloodless surgical field.

#### 2.2.3. Surgical Procedure

**Figure 2:**
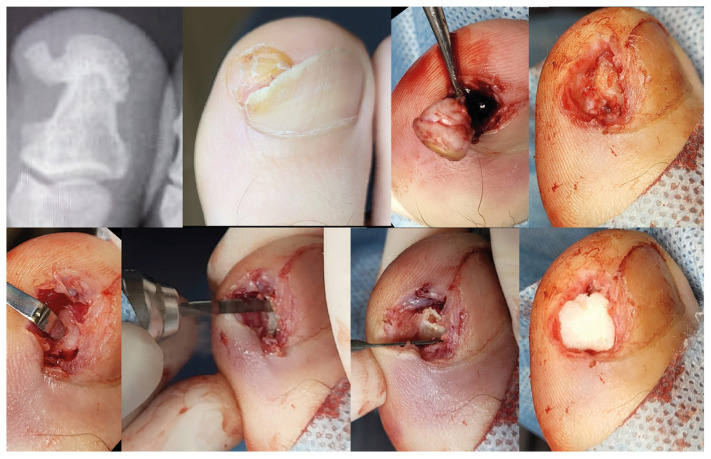
Surgical technique for the resection of subungual osteochondroma/subungual exostosis.

In the first stage, we removed the part of the nail that covers the tumor. Then, we released the tissue around the nail along the SE, down to the bone phalanx. After releasing it, we removed the main fragment of the tumor using a bone nibbler or bone cutter. We exposed the base of the growth attached to the phalanx, trying to find a healthy phalanx cortex on each side. Since June 2020, we have additionally used a small burr to remove excess growth tissue down to the level of the phalanx cortex. Afterward, we packed the defect area with a hemostatic gelatin sponge (Figure 2). All removed SO/SE specimens were sent for histopathological examination.

A sterile dressing was placed; this was removed by the patient after 48 h. The patient changed subsequent dressings every 24 h. The average duration of surgery was approximately 25–40 min, including the waiting time for anesthesia.

#### 2.2.4. After Surgery

Patients were discharged home about 30 min after surgery. They received an information card and the same wound recommendations as in our previously described articles on ingrown toenail surgery [23,24].

### 2.3. Macroscopic Assessment

The nail defect was assessed before the surgery and during the procedure using preoperative and intraoperative photos. With the aid of the Irfanview software (http://www.irfanview.com (accessed on 19 August 2023)), a mask divided into 16 equal rectangles was overlaid on the nail image, and the number of nail defects caused by the tumor was measured.

### 2.4. Histopathological Assessment

**Figure 3:**
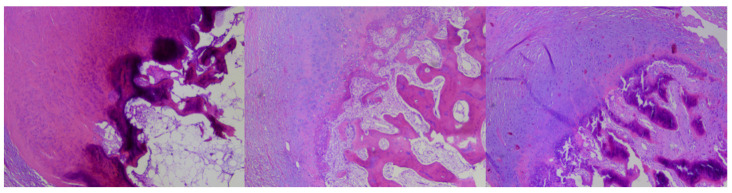
Three types of changes from the examined series: subungual osteochondroma (SO)—trabecular bone covered with hyaline cartilage (**left**), subungual exostosis (SE)—covered with fibrocartilage (**right**) and mixed type covered with hyaline-fibrocartilage (**middle**).

The sections were then transferred to Menzel (Thermo Scientific, Braunschweig, Germany) glass slides and stained with hematoxylin and eosin using the Leica ST5020 automatic stainer. After applying cover slips, the material was evaluated under a light microscope (Leica DM3000) by a specialist pathomorphologist with 8 years of experience in histological slide assessment (Figure 3). 

### 2.5. Clinical Assessment

To assess the surgical procedure and obtain clinical information, we used a questionnaire. Participants were asked to complete the Surgical Satisfaction Questionnaire (SSQ) [25,26]. This instrument consists of eight items related to the patient’s personal satisfaction with the procedure [23].

Pain was assessed twice on an 11-point Visual Analogue Scale (VAS). The first measurement was taken before surgery and another measurement was obtained while collecting data on the satisfaction with the procedure. 

### 2.6. Statistical Analysis

Statistica 13.0 software (StatCorp., College Station, TX, USA) and Microsoft Excel 2016 were used for the analysis. Descriptive values of variables were expressed as means ± standard deviations or medians (minimum–maximum). Dichotomous data were presented as counts and frequencies. The analyses performed showed that the examined parameters did not have a normal distribution. A logit regression model was used for the analysis of odds ratios (OR) with a 95% confidence interval (CI) to identify factors associated with a worse satisfaction effect and recurrence. We used the *p*-values of the likelihood ratio (LR) test. Statistical significance was set at *p* < 0.05.

## 3. Results

### 3.1. Baseline Characteristics

The average follow-up time was 38.7 months. The average age of the patients was 29.6 years, with a standard deviation (SD) of 16.7. The majority of the patients were female, accounting for 73% of the total, while males constituted 27%. The mean duration of symptoms before surgery was 14.1 months (SD = 13). The big toe was the most commonly treated digit, representing 78.4% of the cases. Other affected fingers included the fourth toe (6.8%), followed by the second toe (5.4%), fifth toe (2.7%), third toe (2.7%), fourth finger on the hand (2.7%) and second finger on the hand (1.4%). Left-sided surgeries accounted for 62.2% of the cases. The majority of the cases were located on the medial side (73%), followed by the lateral side (36.5%) and the central area (2.7%). Trauma/injury was the most common risk factor (24.3%), followed by long-term pressure (18.9%) and chronic infection (8.1%). A significant portion of the cases (55.4%) had no identifiable risk factors. Dermatological treatment and surgical removal of the nail were the most common previous treatments, each accounting for 18.9% of the cases. Antibiotic therapy (4.1%), podological/podiatric treatment (10.8%) and biopsy (1.4%) were also noted. A considerable proportion of the patients (51.4%) had not received any previous treatment. The mean assessment score for nail loss before surgery was 4.4, with a standard deviation of 2.2. The median score was 4, ranging from 1 to 10. On average, there was an intraoperative assessment of tissue loss of 6.3 (SD = 2.3). The mean VAS score for pain before surgery was 4.6, with a standard deviation of 2.8. The median score was 5, ranging from 0 to 10 (Table 1).

### 3.2. Patient Satisfaction

The majority of participants (92%) reported being satisfied with pain control in the hospital after surgery. Similarly, a high percentage (90%) expressed satisfaction with pain control when they returned home. The vast majority of participants (94%) reported satisfaction with the amount of time it took them to return to their daily activities, including housework and social activities. The satisfaction level regarding the time taken to return to work was also high (94%). A majority of participants (86%) expressed satisfaction with the amount of time it took them to return to their normal exercise routine. The majority of participants (90%) reported being satisfied with the results of their surgery. When asked if they would have the surgery again, a significant percentage (84%) responded positively. Most participants (90%) expressed satisfaction with the esthetic results of the surgery. A majority of participants (82%) reported satisfaction with the postoperative scar (Table 2). 

**Figure 4:**
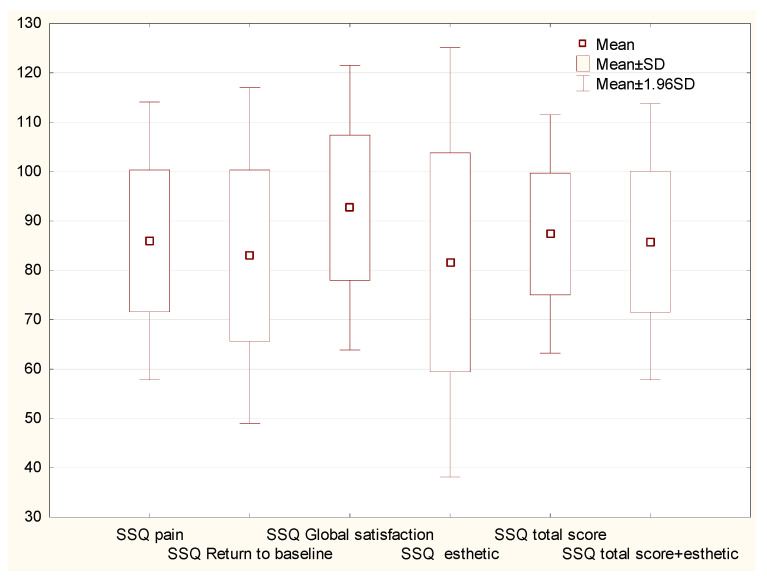
Postoperative responses of patients who underwent surgical treatment for surgical removal of SO/SE. Graph of the mean values and standard deviations of the answers to questions regarding satisfaction with the surgery. Abbreviations: SSQ, Surgical Satisfaction Questionnaire; SD, standard deviation.

The SSQ pain variable had a mean score of 86.3 ± 14.3. In the SSQ Return to baseline variable, the mean score was 83.2 ± 17.2. The SSQ total score had a mean of 87.6 ± 12.3. The SSQ total score variable had a mean score of 86 ± 14.2 (Figure 4). 

Recurrence was the most significant factor of dissatisfaction with treatment (odds ratios 16,286). Age < 25 yo and VAS after surgery showed statistically significant associations (*p* < 0.05) with worse satisfaction (SSQ < 80), with odds ratios of 5.75 and 7994, respectively. The big toe, right side had high OR but was not statistically significant (Table 3). 

Other variables, such as duration of symptoms, involvement of the risk factors, previous treatment, pre-operative assessment of nail loss, intraoperative tissue loss, VAS scores before and after surgery, observation time, burr usage and post-surgery care did not exhibit statistically significant associations with worse satisfaction (Table 3).

### 3.3. Recurrence

**Figure 5:**
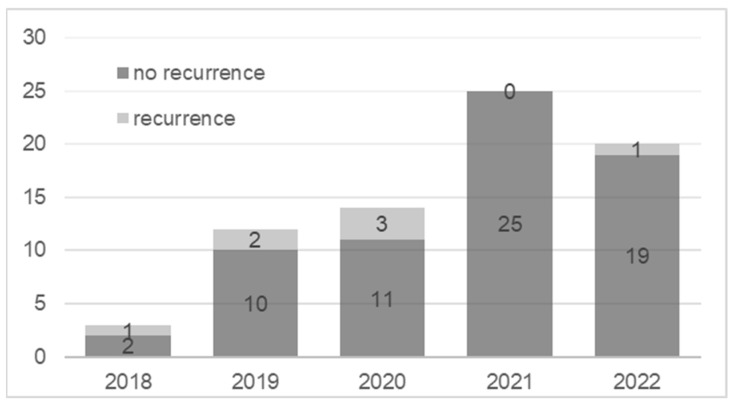
Number of patients (in years) with and without recurrence.

Recurrence of the lesion occurred in seven cases (9.5%). In all cases, reoperations were performed with successful outcomes and no further recurrence. The distribution of cases and recurrences in individual years showed that most recurrences were found in the first year of surgical treatment. Only one case was reported in 2022 (a young boy) and none were reported in 2021 (Figure 5). 

Age < 18 showed a statistically significant association with recurrence, with an odds ratio of 6.806 (*p* = 0.03). Worse satisfaction (SSQ < 80) was strongly associated with recurrence (OR 16.286, *p* = 0.023). Longer observation time increased the likelihood of recurrence (OR 1.062, *p* = 0.043). No burr showed no significant association with recurrence (*p* = 0.093), with an odds ratio of 3922 (Table 4).

### 3.4. Complications

**Figure 6:**
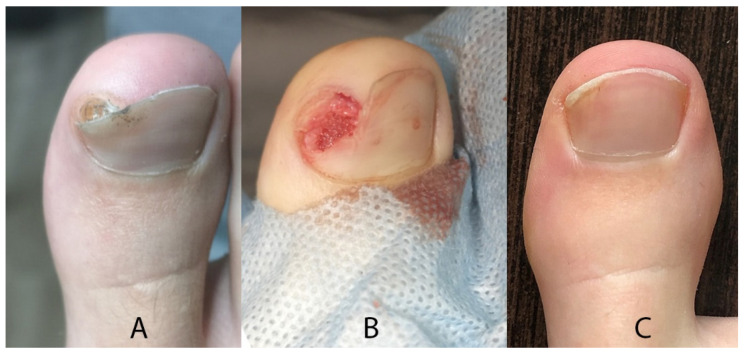
Clinical outcomes of treatment (**A**) before (**B**) intraoperatively and (**C**) after surgery.

Complication occurred in one patient in the form of delayed healing. The main issue stemmed from infrequent podiatric visits post-surgery and the lack of wound cleansing. Ultimately, a favorable final outcome was achieved. The most common cosmetic complaint reported in patients was onycholysis, which occurs in about 70% of the cases (Figure 6).

## 4. Discussion

There is a debate on whether SE and SO represent the same clinical entity but with histopathological variations. Based on previous research, we chose to treat the assessment of SO and SE as a single group, as done by other authors of recent studies on this subject, due to the absence of differences in therapeutic management and outcomes [1,13,27]. 

There were no statistically significant differences between SO and SE in age, gender, duration of illness or presence of pain [3].

Most of the publications on these subungual tumors are based on single clinical cases or small groups of patients. Only 6 publications had a group of more than 50 patients [7]. Some articles did not have a histopathological evaluation and concerned changes in the hypertrophy of the distal phalanx, which we described as surgical technique and treatment effectiveness in our previous article [24].

In this retrospective study, we analyzed the clinical features, treatment outcomes, and recurrence rates of 74 patients with surgically removed SO or SE. The patients were followed up for an average of 38.7 months. The mean age at diagnosis was 29.6 years, and the male-to-female ratio was 2.7:1. The most common location was the big toe (78.4%).

A previous study showed that the average age was 25 (18.75) for SE and 17 for SO, while the M:F ratio was close to 1:1. In another study, the SE population of children and adults had a mean age of 20 (9–63) while the M:F ratio was close to 1:1 [4]. In a meta-analysis of an SE population, the mean age was higher (34.2 years) and the male-to-female ratio showed a predominance of females [7]. Another study of SO showed that the age was nearly 10.7 years and the M:F ratio was 1:1, while a study of SE had an age of 28.4 years [5]. 

Separating our pediatric population with SO/SE (9–17 years old), we had 23 patients (31%) with a mean age of 13.1 years and a gender ratio (M:F) of 13:10. DaCambra et al. reported a 25% pediatric population with SE but used the search terms “subungual exostoses/exostosis” and “subungual osteochondroma” [3]. In other pediatric patients with SO, the sex ratio was the same while the mean age was 11.13 years old [27]. In an SE population, the median age of the patients was 9 years (4–17 years), with a significantly higher proportion of boys (4:1) [22].

All studies showed a predominance of the defect in the big toe [7,27]. In children populations with SE, the defect was located in the big toe in 75% of the cases and the second toe in 20% of the cases [4]. In a pediatric population, SE was located in the big toe in 55% of cases, in the second or third toes in 30% of the cases and the distal phalanxes of the fingers in only 7.5% of the cases—twice as often on the right side [22]. The most frequent locations of SO in children were the first toe (86.8%) and the right lower limb (56.5%) [27].

The recurrence rates reported in the literature range from 5% [22,27] to 14.5% [15]. DaCambra et al. conducted a systematic review of 16 articles on subungual exostosis of the toes with recurrence rates ranging from 0% to 25%, with a mean of 8.3% [3]. The rate in our study was 9.5%.

Understanding the clinical factors that influence treatment outcomes and the recurrence of surgically removed protruded SE/SO is crucial for effective management and patient care. Interestingly, our findings indicate that certain clinical factors such as duration of symptoms, pain before surgery, the size of the tumor and the size of the destruction of the nail plate did not have a significant impact on the recurrence rates of SE/SO. 

We have shown that dissatisfaction is significantly correlated with recurrence. 

Interestingly, we have shown that younger age is conducive to recurrence and poorer treatment outcomes of SE/SO. Younger age at diagnosis may reflect higher growth potential or genetic predisposition to the tumor [1,15]. In pediatric populations, the factors that may contribute include incomplete excision of the tumor, leaving residual bone or cartilage tissue. This may be associated with a less distinct border separating the tumor from the cartilaginous dorsal surface of the distal phalanx. Additionally, the small size of the phalangeal structures also poses a difficulty. This was the probable cause of one recurrence in a child in 2022.

Various surgical techniques for subungual exostosis (SE) involving direct dorsal incision and mass excision have been reported. Nail bed repair methods differ, ranging from partial to total matrixectomy [19,28,29,30,31]. In these techniques, surgical approaches are usually performed via direct dorsal surgical incision. After the mass excision, the surgical approach varies vis-à-vis repair of the nail bed. Goktay et al. reported that the bone rongeur cuts the lesion into fragments and that using a rongeur alone may not completely remove the lesion. The lesion can be cut off from the base in one attempt using nail scissors, and cleaning the base of the lesion with a rongeur or curette seems sufficient to cure SE and SO. Lack of curettage seems to be closely associated with the recurrence of lesions [15]. 

The results of this study demonstrate that surgical resection using a dorsal approach is an effective treatment for SE/SO. The high level of patient satisfaction (85%) and positive cosmetic outcomes (nearly 80% of patients rated the cosmetic effect as good or very good) highlight the success of this surgical technique in improving patients’ quality of life. Our results are in agreement with previous studies that reported high satisfaction rates and low complication rates after surgical treatment of SO and SE [3,15,22,32,33,34]. Surgical resection of SE in the fingers is typically curative, with a small risk of recurrence [5].

The use of a burr to clean the surface of the distal phalanx after tumor removal could have contributed to recurrence, although this was not statistically significant. Based on the analysis of relapse cases over the years, we started using burr routinely in 2021; this significantly reduced the number of recurrences. In our opinion, the use of a burr in the surgical procedure likely contributes to its efficacy. The burr allows for precise and complete removal of the tumor, minimizing the risk of residual growth and recurrence. 

Further analyses focused on factors influencing the results of satisfaction with SE/SO surgical treatment. We observed that age < 25 and VAS after surgery showed statistically significant associations with worse satisfaction (SSQ < 80) and other variables such as duration of symptoms, involvement of risk factors, previous treatment, pre-operative assessment of nail loss and intraoperative tissue loss. The most common cosmetic complaint reported in patients was onycholysis, which occurred in about 70% of the cases. This is due to secondary healing of the lesion. In our opinion, it could be due to the lack of podological care after the procedure, lack of cleansing or irregular use of the regenerating preparation until the nail grows back. On the other hand, it can be considered a tumor complication because often the nail is in the air anyway when the tumor pushes the nail plate out before the procedure. The postoperative care provided to patients likely plays a crucial role in their satisfaction and overall treatment outcome. We agree with creating a balance between maximally effective lesion removal and minimal tissue damage to achieve a good cosmetic effect in the surgical approach to SE and SO [15]. Exploring the potential of regenerative medicine approaches, such as stem cell therapy or tissue engineering, in the treatment of onycholysis after subungual exostosis and subungual osteochondroma could be a promising path. These approaches aim to regenerate damaged tissues and promote secondary healing, potentially offering new therapeutic options in the future [35]. 

The presented data constitute the first comprehensive assessment of the impact of clinical factors on the outcome of treatment of subungual tumors and patient satisfaction. They also constitute the basis for further research on the correlations between factors and recurrences.

The limitations of this study include its retrospective design, single-center setting, the inclusion of children and adult patients and the use of different treatments (the use of burr or not). In addition, the treatment satisfaction questionnaires used present a subjective assessment of patients. Another limitation is the lack of definitive clinical, histopathological and radiological differentiation between SO and SE. Recent studies indicate that ultrasounds are promising [16,17]. Therefore, further prospective, multicenter and long-term studies are needed to confirm our findings and explore other factors that may affect the treatment outcome and recurrence of SO and SE.

## 5. Conclusions

In conclusion, the findings of this study support the use of the dorsal approach and burr under digital anesthesia for surgical resection of SE/SO. The procedure demonstrated high patient satisfaction and favorable cosmetic outcomes. Moreover, the lack of significant influence from various clinical factors on recurrence rates suggests that this surgical technique is reliable regardless of patient demographics or symptom duration. Further research is warranted to validate these findings and to explore potential refinements in surgical management to enhance outcomes even further. 

## Figures and Tables

**Table 1:** Characteristics of the study group (N = 74).

Parameter		Results
Age (years)	Mean ± SD	29.6 ± 16.7
	Median (min–max)	25 (7–74)
Sex	Female	54 (73%)
	Male	20 (27%)
Duration of symptoms (months)	Mean ± SD	14.1 ± 13
	Median (min–max)	11 (1–60)
Toes operated on	big toe	58 (78.4%)
	toe IV	5 (6.8%)
	toe II	4 (5.4%)
	toe V	2 (2.7%)
	toe III	2 (2.7%)
	finger IV (hand)	2 (2.7%)
	finger II (hand)	1 (1.4%)
Side	Left	46 (62.2%)
	Right	28 (37.8%)
Localization	Lateral	19 (25.7%)
	Medial	54 (73%)
	Central	11 (1.4%)
Localization	Distal	42 (56.8%)
	Proximal	32 (43.2%)
Risk factors	trauma/injury	18 (24.3%)
	long-term pressure	14 (18.9%)
	chronic infection	6 (8.1%)
	none	41 (55.4%)
Previous treatment	dermatological	14 (18.9%)
	Surgical removal of the nail	14 (18.9%)
	antibiotic therapy	3 (4.1%)
	podological/podiatrics	8 (10.8%)
	biopsy	1 (1.4%)
	Not treated	38 (51.4%)
Pre-operative macroscopic assessment of nail loss	Mean ± SD	4.4 ± 2.2
	Median (min–max)	4 (1–10)
Intraoperative assessment of tissue loss	Mean ± SD	6.3 ± 2.3
	Median (min–max)	6 (2–12)
VAS before surgery	Mean ± SD	4.6 ± 2.8
	Median (min–max)	5 (0–10)
Burr usage	No	21 (28.4%)
	Yes	53 (71.6%)

SD, standard deviation.

**Table 2:** Participant ratings on the surgical satisfaction questionnaire after surgery (n = 50).

Item	Response	Participant Rating
1. How satisfied are you with how your pain was controlled in the hospital after surgery?	Satisfied	46 (92%)
	Neutral/unsatisfied	4 (8%)
2. How satisfied are you with how your pain was controlled when you returned home after surgery?	Satisfied	45 (90%)
	Neutral/unsatisfied	5 (10%)
3. How satisfied are you with the amount of time it took for you to return to your daily activities, for example housework or social activities outside the home?	Satisfied	47 (94%)
	Neutral/unsatisfied	3 (6%)
4. How satisfied are you with the amount of time it took for you to return to work?	Satisfied	47 (94%)
	Neutral/unsatisfied	3 (6%)
5. How satisfied are you with the amount of time it took for you to return to your normal exercise routine?	Satisfied	43 (86%)
	Neutral/unsatisfied	7 (14%)
6. How satisfied are you with the results of your surgery?	Satisfied	45 (90%)
	Neutral/unsatisfied	5 (10%)
7. Looking back, if you “had to do it all over again” would you have the surgery again?	Yes	42 (84%)
	Maybe	4 (8%)
	Unsure/do not think so/no	4 (8%)
8. Would you recommend this surgery to someone else?	Yes	47 (94%)
	Maybe	2 (4%)
	Unsure/do not think so/no	1 (2%)
9. How satisfied are you with the esthetic results after surgery?	Satisfied	45 (90%)
	Neutral/unsatisfied	5 (10%)
10. How satisfied are you the current appearance of the problem?	Satisfied	37 (74%)
	Neutral/unsatisfied	13 (26%)
11. How satisfied are you the appearance of the postoperative scar?	Satisfied	41 (82%)
	Neutral/unsatisfied	9 (18%)

**Table 3:** Estimated coefficients for a logit regression model for worse satisfaction effect SSQ < 80.

Variables	OR (95% CI)	*p*-Value
Age < 25 yo	5.75 (1.076–30.721)	0.041
Sex: Male	2.222 (0.512–9.647)	0.286
Duration of symptoms (months)	1 (0.955–1.047)	0.993
Big toe	4.5 (0.513–39.438)	0.174
Side: Right	2.9 (0.692–12.154)	0.145
Risk factors	1.941 (0.472–7.988)	0.358
No Previous treatment	0.95 (0.237–3.812)	0.942
Pre-operative macroscopic assessment	0.88 (0.641–1.207)	0.426
Intraoperative amount of tissue loss	1.019 (0.752–1.38)	0.906
pain before surgery (VAS)	1.113 (0.865–1.434)	0.405
pain after surgery (VAS)	7.994 (2.12–30.141)	0.002
Observation time	1.04 (0.986–1.097)	0.149
Recurrence	16.286 (1.474–179.97)	0.023
No burr	1.5 (0.357–6.308)	0.58
podological/podiatrics care after surgery	2 (0.37–10.801)	0.42

Abbreviations: OR, odds ratio; CI, confidence interval; VAS, Visual Analogue Scale; SD, standard deviation; SSQ, Surgical Satisfaction Questionnaire.

**Table 4:** Estimated coefficients for a logit regression model of recurrence.

Variables	OR (95% CI)	*p*-Value
Age < 18 yo	6.806 (1.211–38.255)	0.03
Sex: Male	1.089 (0.194–6.121)	0.923
Duration of symptoms (months)	0.985 (0.92–1.056)	0.676
Big toe	1.731 (0.193–15.52)	0.624
Side: Right	4.783 (0.86–26.593)	0.074
Risk factors	2.903 (0.526–16.031)	0.221
No Previous treatment	0.344 (0.062–1.902)	0.221
Pre-operative macroscopic assessment of nail loss	0.963 (0.669–1.385)	0.838
Intraoperative amount of tissue loss	1.175 (0.856–1.615)	0.319
pain before surgery (VAS)	0.916 (0.635–1.323)	0.641
pain after surgery (VAS)	1.297 (0.767–2.195)	0.332
Observation time	1.062 (1.002–1.126)	0.043
Worse satisfaction SSQ < 80	16.286 (1.474–179.97)	0.023
No burr	3.922 (0.796–19.325)	0.093
No podological/podiatrics care after surgery	2.462 (0.313–19.377)	0.392

Abbreviations: OR, odds ratio; CI, confidence interval; VAS, Visual Analogue Scale; SD, standard deviation; SSQ, Surgical Satisfaction Questionnaire.

## Data Availability

The data presented in this study are available on request from the corresponding author.
